# A side-by-side evaluation of [^18^F]FDOPA enantiomers for non-invasive detection of neuroendocrine tumors by positron emission tomography

**DOI:** 10.18632/oncotarget.27184

**Published:** 2019-10-08

**Authors:** Athira Narayan, Yu Yan, Ala Lisok, Mary Brummet, Martin G. Pomper, Wojciech G. Lesniak, Robert F. Dannals, Vanessa F. Merino, Babak Behnam Azad

**Affiliations:** ^1^Russell H. Morgan Department of Radiology and Radiological Science, Johns Hopkins School of Medicine, Baltimore, MD, USA

**Keywords:** FDOPA, PET, neuroendocrine tumors, Fluorine-18, molecular imaging

## Abstract

Neuroendocrine tumors (NETs) are an extremely heterogenous group of malignancies with variable clinical behavior. Molecular imaging of patients with NETs allows for effective patient stratification and treatment guidance and is crucial in selection of targeted therapies. Positron emission tomography (PET) with the radiotracer L-[^18^F]FDOPA is progressively being utilized for non-invasive *in vivo* visualization of NETs and pancreatic β-cell hyperplasia. While L-[^18^F]FDOPA-PET is a valuable tool for disease detection and management, it also exhibits significant diagnostic limitations owing to its inherent physiological uptake in off-target tissues. We hypothesized that the D-amino acid structural isomer of that clinical tracer, D-[^18^F]FDOPA, may exhibit superior clearance capabilities owing to a reduced *in vivo* enzymatic recognition and enzyme-mediated metabolism. Here, we report a side-by-side evaluation of D-[^18^F]FDOPA with its counterpart clinical tracer, L-[^18^F]FDOPA, for the non-invasive *in vivo* detection of NETs. *In vitro* evaluation in five NET cell lines, including invasive small intestinal neuroendocrine carcinomas (STC-1), insulinomas (TGP52 and TGP61), colorectal adenocarcinomas (COLO-320) and pheochromocytomas (PC12), generally indicated higher overall uptake levels of L-[^18^F]FDOPA, compared to D-[^18^F]FDOPA. While *in vivo* PET imaging and *ex vivo* biodistribution studies in PC12, STC-1 and COLO-320 mouse xenografts further supported our *in vitro* data, they also illustrated lower off-target retention and enhanced clearance of D-[^18^F]FDOPA from healthy tissues. Cumulatively our results indicate the potential diagnostic applications of D-[^18^F]FDOPA for malignancies where the utility of L-[^18^F]FDOPA-PET is limited by the physiological uptake of L-[^18^F]FDOPA, and suggest D-[^18^F]FDOPA as a viable PET imaging tracer for NETs.

## INTRODUCTION

Neuroendocrine neoplasms (NENs) are an extremely heterogenous group of malignancies with variable clinical behavior including hormonally active or inactive tumors that range from being well-differentiated and slow growing to poorly differentiated and aggressive [1, 2]. NENs originate from the endocrine glands as well as endocrine islets embedded within grandular tissue and scattered cells in the exocrine parenchyma [3]. While they can occur anywhere in the body, neuroendocrine tumors (NETs) are most commonly observed in the gastrointestinal and bronchopulmonary tracts. Treatment options include surgical resection, for localized disease, and systemic treatments such as chemotherapy, peptide receptor radionuclide therapy (PRRT) and somatostatin analogues for metastatic disease [4-8]. In all cases, however, molecular imaging is heavily utilized for treatment selection and guidance [9-11]. Image-guided therapy in NETs is especially important given the drastic variability in phenotype and functionality of NET subtypes. In addition, imaging allows for more effective patient stratification for targeted therapies, such as PRRTs, by confirming the presence of cell surface receptors, such as somatostatin receptor type 2 (SSTR2).

Positron emission tomography (PET) with the PET radiotracer L-[^18^F]FDOPA, a radiolabeled analogue of the amino acid L-DOPA, is progressively being utilized for non-invasive *in vivo* visualization of NETs and pancreatic β-cell hyperplasia [12-14]. L-[^18^F]FDOPA-PET is particularly effective for the diagnosis and staging of pheochromocytomas and paragangliomas as well as differentiating between focal and diffuse pancreatic disease in hyperinsulinemic newborns. The underlying principle behind amino acid-based imaging is the enhanced unabated consumption of nutrients by cancer cells for sustained growth and proliferation. The higher requirement for amino acids is then alleviated by the upregulation of amino acid transporters located on the plasma membrane, which actively facilitate amino acid movement. The L-type amino acid transporter 1 (LAT1), which mediates L-[^18^F]FDOPA uptake, is highly upregulated in numerous NETs including pheochromocytoma, paraganglioma, medullary thyroid carcinoma (MTC), lung NETs and gastrointestinal carcinomas [15-18]. L-[^18^F]FDOPA is currently in clinical trials for detection of MTCs, carcinoid tumors, pheochromocytomas, paragangliomas, insulinomas and neuroblastomas (NCT02431715).

While L-[^18^F]FDOPA-PET is a valuable tool for disease detection and management, it also exhibits significant diagnostic limitations owing to its inherent physiological biodistribution [19]. For instance, L-[^18^F]FDOPA-PET exhibits intense focal uptake in the gallbladder and, in some cases, the bile tract, which could resemble an intestinal tumor or NET-derived hepatic metastasis. Moderate L-[^18^F]FDOPA uptake is also observed in the striatum, liver, and myocardium. Because L-[^18^F]FDOPA clearance is primarily via renal excretion, the intense uptake of this tracer in the kidneys could also mask pathological uptake in the pancreas, particularly in the pancreatic tail, which may be normalized by signals from the left kidney, adrenals and ureters [20-22]. L-[^18^F]FDOPA also exhibits intense but variable uptake in the pancreas, which may be confused for a para-aortic pathologic lesion [23]. Because L-[^18^F]FDOPA physiological uptake is at least partially a result of its enzymatic metabolism, premedication with enzyme inhibitors can reduce physiological L-[^18^F]FDOPA uptake in some healthy tissues [24]. For instance, normal pancreatic uptake can be largely prevented by premedication with Carbidopa [25]. However, the use of enzyme inhibitors can also lower tumor uptake levels in some cases, such as for islet cell tumors, β-cell hyperplasia and insulinomas, and as such is not a universal solution to lowering the physiological uptake of L-[^18^F]FDOPA [26]. Cumulatively, the above findings are particularly problematic for some NETs, especially smaller tumors in the midgut region, which may be difficult to detect by L-[^18^F]FDOPA-PET. Utilization of alternate strategies to lower L-[^18^F]FDOPA uptake in healthy tissues will circumvent the limitations of L-[^18^F]FDOPA-PET, thus making it a more effective diagnostic tool applicable to a larger number of malignancies.

Here, we report the evaluation of a structural isomer (enantiomer) of L-[^18^F]FDOPA, D-[^18^F]FDOPA, for non-invasive *in vivo* detection of NETs. We hypothesized that because certain enzymes in the FDOPA metabolic pathway, such as aromatic amino acid decarboxylase (AADC), are stereospecific and only recognize naturally occurring L-amino acids, D-[^18^F]FDOPA may exhibit a reduced metabolism and thus lower non-specific uptake in healthy tissues, compared with L-[^18^F]FDOPA. A side-by-side *in vitro* and *in vivo* evaluation of both [^18^F]FDOPA isomers was carried out to assess the differences in pharmacokinetics of these tracers and their ability for non-invasive detection of NETs. To the best of our knowledge, reports on the utility of D-[^18^F]FDOPA are limited to a published abstract on its use for detection of melanomas [27]. The current report is the first comprehensive report showing a side-by-side evaluation of D-[^18^F]FDOPA and its clinical tracer counterpart L-[^18^F]FDOPA in any disease model, and the first report on the evaluation of D-[^18^F]FDOPA, in NETs.

## RESULTS

### Preparation of D- and L-[^18^F]FDOPA

**Figure 1:**
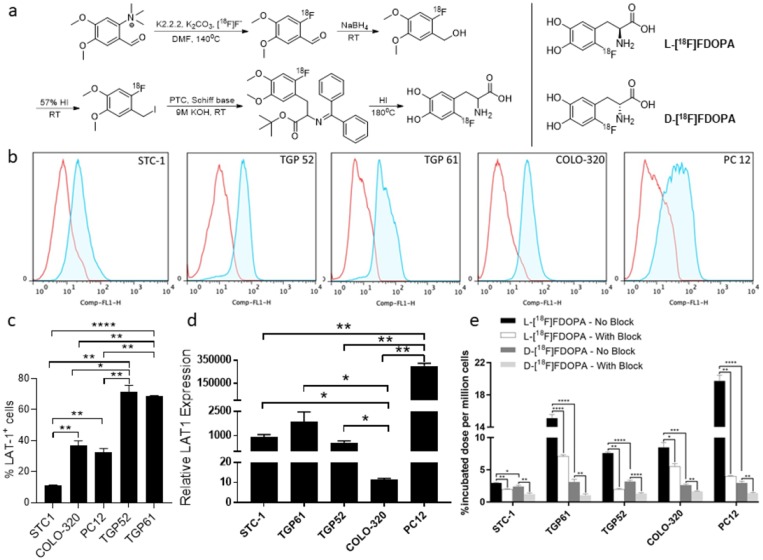
Preparation of [^18^F]FDOPA enantiomers and *in vitro* evaluation. Synthetic route for the preparation of [^18^F]FDOPA enantiomers **(a)**; LAT1 protein expression by flow cytometry **(b-c)** and mRNA expression by RT-PCR **(d)** in NET cell lines; *in vitro* uptake assays with L-[^18^F]FDOPA and D-[^18^F]FDOPA **(e)** ; ^*^ p<0.05, ^**^ p<0.01, ^***^ p<0.001; ^****^ p<0.0001.

Synthesis of both FDOPA enantiomers followed established literature reports as outlined in Figure 1a [28]. This synthesis entailed the nucleophilic radiofluorination of a commercially available trimethyl ammonium precursor, followed by reduction, halogenation, enantioselective alkylation and subsequent deprotection steps as reported in the above referenced study. L-[^18^F]FDOPA was prepared over 70mins with an average non-decay corrected radiochemical yield of 40±10% (n=15). D-[^18^F]FDOPA was synthesized over 90 mins with an average non-decay corrected radiochemical yield of 18±5% (n=15). Radiochemical purities and enantiomeric excess for both D- and L-FDOPA enantiomers were >97% as determined by chiral, reverse phase, high performance liquid chromatography (RP-HPLC). Final formulations were stable and did not show any signs of radiolysis or degradation up to 9hrs post synthesis. Average specific activities for both radiotracers were comparable and found to be 274±70MBq/μmol (7400±1890mCi/μmol).

### *In vitro* evaluation of [^18^F]FDOPA enantiomers in NET cell lines

NET cell lines representing invasive small intestinal neuroendocrine carcinomas (STC-1), insulinomas (TGP52 and TGP61), colorectal adenocarcinomas (COLO-320) and pheochromocytomas (PC12) were assessed for LAT1 protein (Figure 1b-c) and mRNA (Figure 1d) expression by flow cytometry and RT-PCR, respectively. LAT1 mRNA was also detected in all NETs with the highest levels observed in PC12 cells and lowest levels in COLO-320 cells.

Both [^18^F]FDOPA enantiomers were initially assessed *in vitro* in all NET cells. Obtained results indicated variable uptake of both tracers in all cell lines (Figure 1e). Blockade with the LAT1 inhibitor, BCH, consistently reduced the uptake of both enantiomers, thereby supporting a LAT1 mediated mechanism. L-[^18^F]FDOPA uptake in the studied NETs was generally higher than D-[^18^F]FDOPA, reaching 19.73±0.71% (incubated dose/million cells) in PC12 cells. The lowest uptake of L-[^18^F]FDOPA was observed in the STC-1 cell line (2.97±0.07%). In comparison, the highest uptake of D-[^18^F]FDOPA was observed in TGP52 (3.18±0.19%), TGP61 (3.11±0.41%) and PC12 cells (2.97±0.24%) with no statistically significant differences among those cell lines. Similar to L-[^18^F]FDOPA, the lowest D-[^18^F]FDOPA uptake was observed in STC-1 cells (2.41±0.17%).

### *In vivo* PET-CT and image analysis in NET mouse xenografts

**Figure 2:**
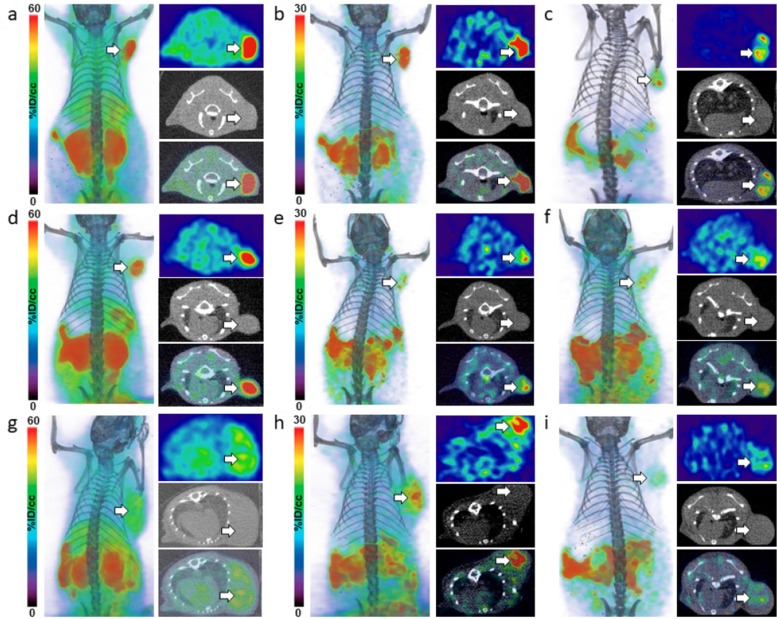
*In vivo* imaging of L-[^18^F]FDOPA in NET mice. PET-CT imaging of L-[^18^F]FDOPA in NSG mice bearing PC12 **(a-c)**, STC-1 **(d-f)** and COLO-320 xenografts **(g-i)**; after injection of 9MBq (250μCi) of L-[^18^F]FDOPA, PET images were acquired at 30min **(a, d, g)** and 120min **(b, e, h)** time points in each NET mouse model; *in vivo* blocking was also carried out in each model with panels **(c, f, i)** showing representative PET-CT images 120min p.i.; dorsal coronal (left) and transverse (PET - top, CT - middle and PET-CT fused - bottom) images are shown for each mouse; white arrows indicate tumors.

**Figure 3:**
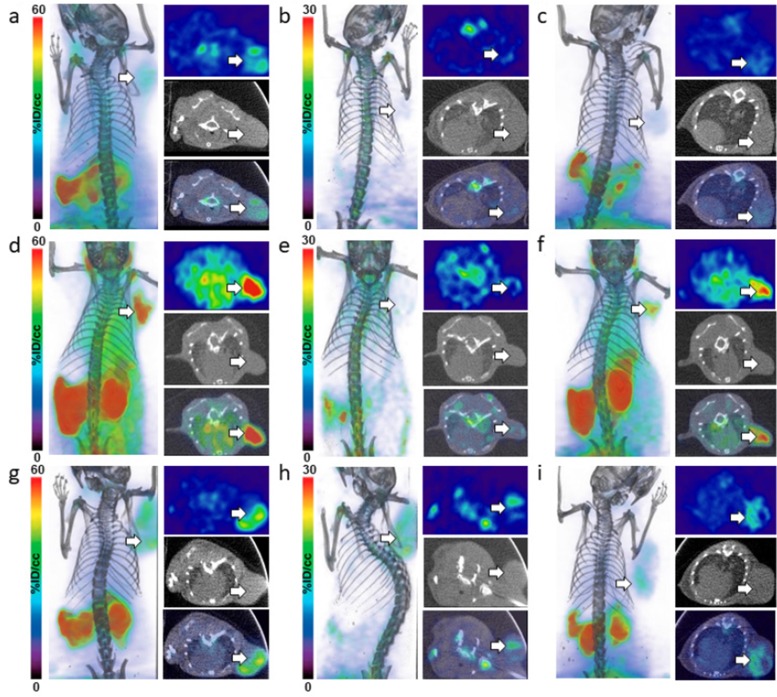
*In vivo* imaging of D-[^18^F]FDOPA in NET mice. PET-CT imaging of D-[^18^F]FDOPA in NSG mice bearing PC12 **(a-c)**, STC-1 **(d-f)** and COLO-320 xenografts **(g-i)**; after injection of 9MBq (250μCi) of D-[^18^F]FDOPA, PET images were acquired at 30min **(a, d, g)** and 120min **(b, e, h)** time points in each NET mouse model; *in vivo* blocking was also carried out in each model with panels **(c, f, i)** showing representative PET-CT images 120min p.i.; dorsal coronal (left) and transverse (PET - top, CT - middle and PET-CT fused - bottom) images are shown for each mouse; white arrows indicate tumors.

**Figure 4:**
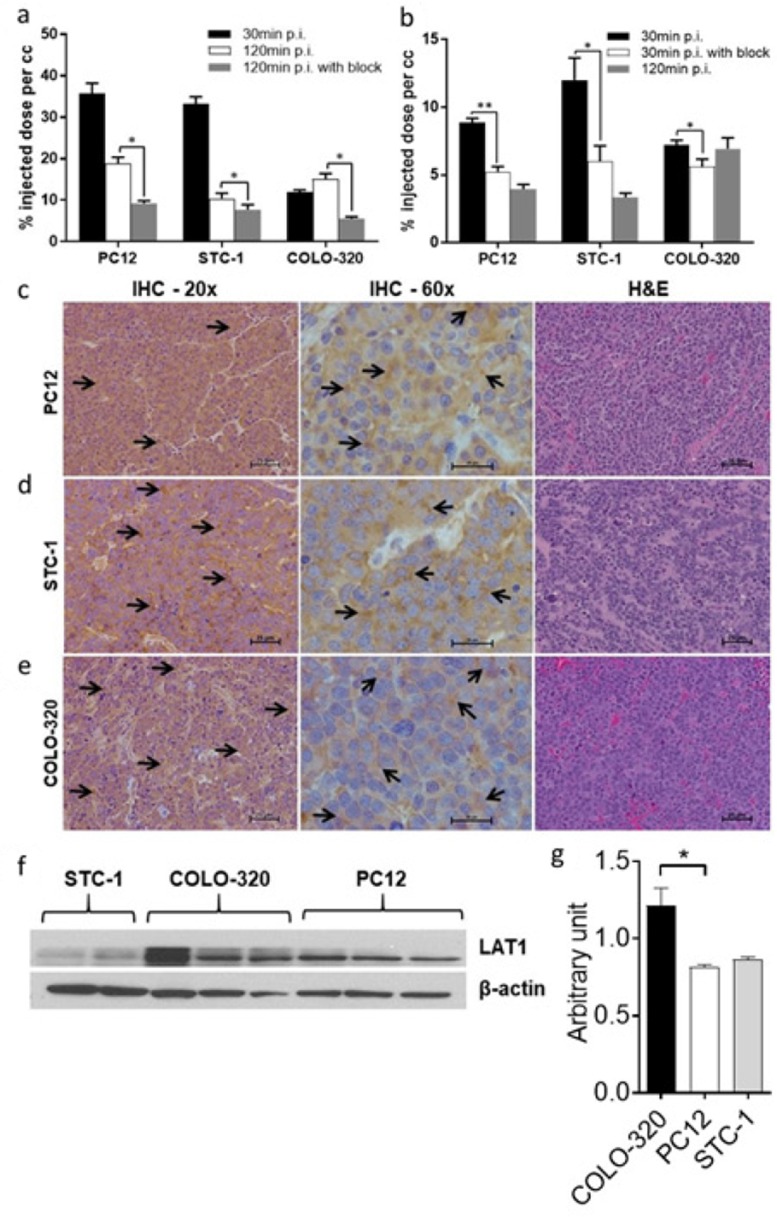
Analysis of PET images and excised tumor tissues. Quantitative analysis of acquired *in vivo* PET images 30min and 120min after tail vein injection of 9MBq (250μCi) L-[^18^F]FDOPA **(a)** or D-[^18^F]FDOPA **(b)** with or without blocking with 10meq of the LAT1 inhibitor; results show a reduction in tumor uptake after blocking, indicating a LAT1-mediated uptake of each tracer in all three NET subtypes; IHC and H&E (20x and 60x magnification; scale 25μm) analysis of excised PC12 **(c)**, STC-1 **(d)** and COLO-320 tumors **(e)** further confirming LAT1 expression in the studied NETs; arrows indicate regions of LAT1 immunoreactivity in the tumor tissues; western blot analysis of tumor tissues retrieved from NSG mice, showing LAT1 protein expression (43kDa) in all three NET subtypes **(f)**; quantification of western blot results (n=4) illustrating variable LAT1 expression in all NETs **(g)**; ^*^ p<0.05, ^**^ p<0.01.

To assess the *in vivo* pharmacokinetic profiles of D- and L-[^18^F]FDOPA enantiomers, PC12, COLO-320 and STC-1 cell lines, exhibiting variable LAT1 expression, were used for *in vivo* evaluation. PET-CT imaging was carried out with L-[^18^F]FDOPA and D-[^18^F]FDOPA in NSG mice bearing NET xenografts (Figures 2 and 3). Mice were imaged at 30min and 120min post intravenous injection of each tracer. *In vivo* blocking by BCH resulted in significantly reduced L-[^18^F]FDOPA (Figure 2c, 2f, 2i) and D-[^18^F]FDOPA (Figure 3c, 3f, 3i) uptake levels in all NET mouse models, as also evident by *in vivo* image quantification (Figure 4a-4b), further supporting a LAT1-mediated mechanism. Immunohistochemistry (IHC) and H&E staining (Figure 4c-4e) as well as western blot analysis (Figure 4f-4g) of excised tumor tissues supported LAT1 expression in all NETs, with IHC showing primarily intracellular LAT1 staining. Our IHC results are in agreement with literature reports showing cytoplasmic LAT1 staining in tumor tissues [17],[29],[30]. While, the exact mechanisms for this localization as well as the nature of intracellular LAT1 have not been resolved, it has been suggested that cytoplasmic LAT1, which also appears to be disease dependent, may either have a different yet undiscovered function or may be non-functional entirely, compared to cell surface LAT1 transporters [31],[32].

In line with our *in vitro* findings, *in vivo* PET-CT imaging indicated generally higher L-[^18^F]FDOPA tumor uptake, compared to D-[^18^F]FDOPA, in all three NET mouse models. L-[^18^F]FDOPA was particularly the superior tracer in PC12 mice (Figure 2a, 2b) with quantitative PET image analysis indicating a tumor uptake of 35.73±2.50%ID/cc, 30min post tracer injection (Figure 4a). This uptake decreased to 18.82±1.55%ID/cc, 120min post tracer injection. In comparison, D-[^18^F]FDOPA uptake in PC12 tumors (Figure 3a, 3b) was 8.87±0.31%ID/cc and 3.96±0.34%ID/cc at the same time points, respectively (Figure 4b). In STC-1 mice, L-[^18^F]FDOPA uptake was 33.29±1.66%ID/cc and 10.38±1.31%ID/cc at 30min and 120min time points (Figure 2d, 2e), compared to 12.01±1.61%ID/cc and 3.34±0.32%ID/cc for D-[^18^F]FDOPA (Figure 3d, 3e), respectively. In COLO-320 mice, L-[^18^F]FDOPA tumor uptake was 11.99±0.50%ID/cc and 15.10±1.30%ID/cc at 30min and 120min (Figure 2g, 2h), compared to 7.20±0.35%ID/cc and 6.93±0.80%ID/cc for D-[^18^F]FDOPA (Figure 3g, 3h), respectively. Interestingly, a slight increase in L-[^18^F]FDOPA uptake was observed in COLO-320 tumors at 120min p.i., which was in contrast to PC12 and STC-1 models where significant tracer clearance from tumors was observed over time. Similarly, D-[^18^F]FDOPA uptake and retention in COLO-320 tumors was maintained up to 120min post tracer administration with no statistically significant clearance from tumors tissues. This is also in contrast with PC12 and STC-1 tumors, which exhibited D-[^18^F]FDOPA tumor clearance during the same time period.

While L-[^18^F]FDOPA consistently showed higher tumor uptake values, it also exhibited significant off target retention in peripheral tissues, including pancreas, small intestines, stomach, kidneys, spleen and liver, in all three NET mouse models, even 120min post tracer injection. Conversely, D-[^18^F]FDOPA showed much faster clearance from non-specific tissues in all three mouse models within 120min following tracer administration. The superiority of D-[^18^F]FDOPA was particularly evident in COLO-320 mice at the 120min time point, as PET images primarily showed tumor uptake with significant clearance from all other tissues, including pancreas, small intestines, stomach, kidneys, spleen and liver, which act as primary sinks for L-[^18^F]FDOPA.

### *Ex vivo* biodistribution studies

**Figure 5:**
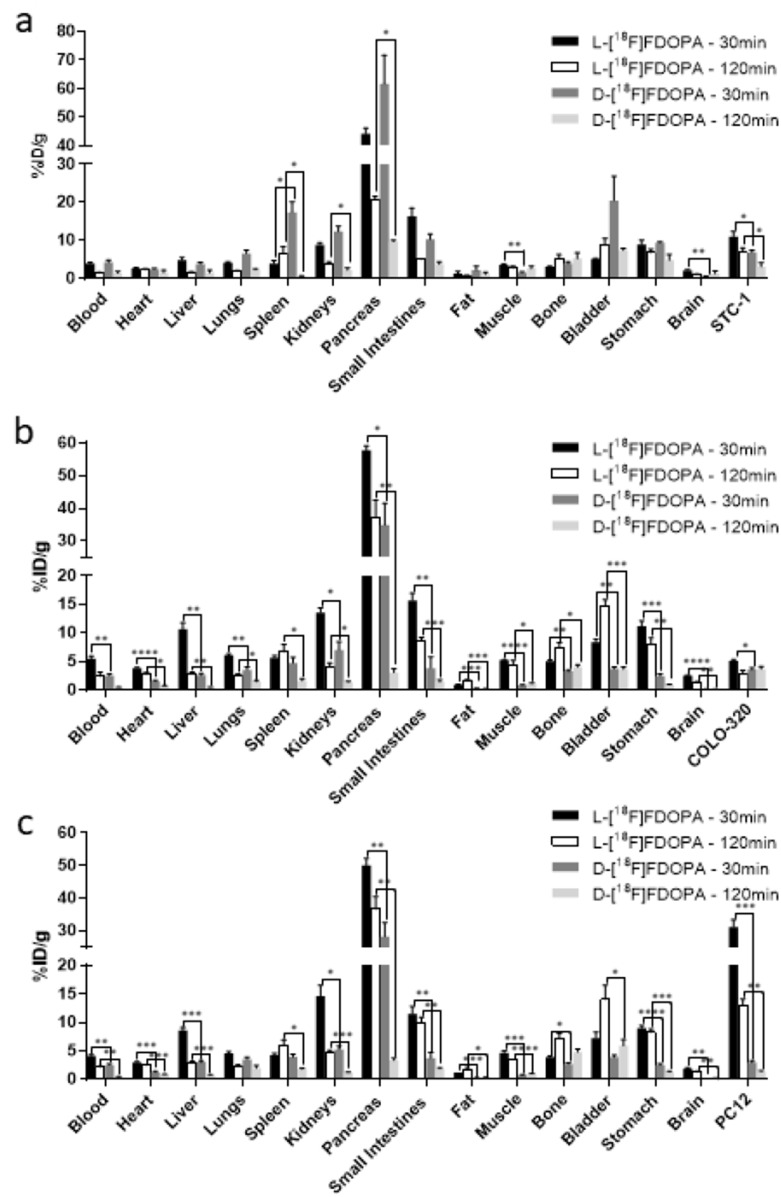
Biodistribution of [^18^F]FDOPA enantiomers in NET models. *Ex vivo* biodistribution in NSG mice bearing STC-1 **(a)**, COLO-320 **(b)** and PC12 **(c)** xenografts; mice were injected with 1.5MBq (40μCi) of either L-[^18^F]FDOPA or D-[^18^F]FDOPA and subsequently sacrificed at 30min and 120min p.i. for tissue retrieval; ^*^ p<0.05, ^**^ p<0.01, ^***^ p<0.001; ^****^ p<0.0001; tissues without ^*^ were not statistically significant.

*Ex vivo* biodistribution studies were carried out at imaging time points (30min and 120min post tracer administration) for a more accurate comparison. Quantified results from these studies have been summarized in Tables 1 and 2 as well as Figure 5. Data from the *ex vivo* biodistribution studies correlated with the obtained PET imaging results, showing higher tumor uptake of L-[^18^F]FDOPA than D-[^18^F]FDOPA in all NETs. At 30min post injection (p.i.), L-[^18^F]FDOPA uptake in STC-1 (Figure 5a), COLO-320 (Figure 5b) and PC12 (Figure 5c) tumors was 11.06±1.29%ID/g, 31.19±2.24%ID/g and 5.15±0.25%ID/g, respectively, decreasing to 6.98±1.12%ID/g, 12.99±2.28%ID/g and 2.96±0.46%ID/g, 120min p.i. Interestingly, the focal increase in COLO-320 tumor uptake seen on PET images was not observed in *ex vivo* biodistribution studies, which was likely owing to the heterogeneous uptake of L-[^18^F]FDOPA in NETs, which when accounting for total tumor mass would result in lower %ID/g values in the biodistribution studies. D-[^18^F]FDOPA uptake in STC-1, PC12 and COLO-320 tumors (Figure 5) was 6.92±0.63%ID/g, 2.95±0.37%ID/g and 3.61±0.34%ID/g, respectively. Correlating with imaging results, at 120min p.i., D-[^18^F]FDOPA uptake decreased in STC-1 (3.15±1.04%ID/g) and PC12 (1.44±0.14%ID/g) tumors while being completely retained in COLO-320 (3.72±0.44%ID/g) tumors.

**Table 1:** Biodistribution of L-[^18^F]FDOPA in NET models

	STC-1				COLO-320				PC12			
**L-[*^18^*F]FDOPA**	*30min*		*120min*		*30min*		*120min*		*30min*		*120min*	
	Average	SEM	Average	SEM	Average	SEM	Average	SEM	Average	SEM	Average	SEM
*Blood*	3.94	0.31	1.47	0.17	5.53	0.43	2.44	0.70	4.14	0.21	2.19	0.17
*Heart*	2.72	0.18	2.34	0.29	3.85	0.14	2.74	0.37	2.96	0.16	2.45	0.18
*Liver*	4.87	0.70	1.73	0.09	10.68	1.13	2.85	0.34	8.59	0.55	3.01	0.19
*Lungs*	4.13	0.34	1.89	0.23	6.11	0.20	2.55	0.33	4.57	0.31	2.43	0.16
*Spleen*	3.98	0.76	6.55	1.88	5.72	0.34	6.72	1.28	4.30	0.32	6.11	0.84
*Kidneys*	8.90	0.54	4.00	0.44	13.60	0.81	4.16	0.62	14.60	1.91	4.84	0.30
*Pancreas*	44.22	1.93	20.71	0.89	57.82	1.40	37.13	5.43	50.08	2.08	36.66	3.81
*Small Intestines*	16.43	2.07	5.08	0.10	15.72	1.28	8.66	0.63	11.50	1.34	10.02	0.91
*Fat*	1.34	0.52	0.78	0.13	0.95	0.06	1.76	0.11	1.25	0.01	1.78	0.35
*Muscle*	3.63	0.23	2.75	0.55	5.27	0.09	4.40	0.81	4.70	0.34	3.48	0.04
*Bone*	3.09	0.10	5.40	0.92	5.05	0.23	7.40	0.93	3.86	0.28	7.19	0.88
*Bladder*	5.15	0.20	8.80	1.76	8.38	0.57	14.73	1.09	7.24	1.10	14.06	2.45
*Stomach*	8.97	1.11	6.83	0.93	11.27	0.88	7.99	1.14	9.04	0.45	8.28	0.56
*Brain*	2.16	0.25	1.07	0.14	2.58	0.08	1.28	0.19	1.90	0.18	1.31	0.15
*Tumor*	11.06	1.29	6.98	1.12	5.15	0.25	2.96	0.46	31.19	2.24	12.99	1.14

*Notes:* *Ex vivo* biodistribution in NSG mice bearing STC-1, COLO-320 and PC12 xenografts after injection with 1.5MBq (40μCi) of L-[^18^F]FDOPA; mice were subsequently sacrificed at 30min and 120min p.i. time points for tissue retrieval; average values and standard errors (SEM) have been shown for each tissue.

**Table 2:** Biodistribution of D-[^18^F]FDOPA in NET models

	STC-1				COLO-320				PC12			
**D-[*^18^*F]FDOPA**	*30min*		*120min*		*30min*		*120min*		*30min*		*120min*	
	Average	SEM	Average	SEM	Average	SEM	Average	SEM	Average	SEM	Average	SEM
*Blood*	4.31	0.60	1.49	0.45	2.58	0.29	0.51	0.04	2.52	0.29	0.48	0.04
*Heart*	2.52	0.22	1.58	0.61	1.57	0.17	0.80	0.05	1.28	0.16	0.84	0.08
*Liver*	3.85	0.38	1.60	0.65	2.65	0.30	0.54	0.04	3.05	0.28	0.73	0.10
*Lungs*	6.63	0.91	2.34	0.31	3.60	0.45	1.52	0.20	3.53	0.41	2.04	0.43
*Spleen*	17.41	2.76	0.61	0.11	4.77	1.03	1.67	0.38	3.99	0.42	1.86	0.14
*Kidneys*	12.32	1.49	2.30	0.54	7.05	1.45	1.44	0.11	5.30	0.56	1.23	0.10
*Pancreas*	61.64	10.01	9.75	0.36	34.93	6.63	3.12	0.68	28.22	4.35	3.42	0.31
*Small Intestines*	10.28	1.41	3.80	0.67	3.89	1.98	1.46	0.34	3.76	0.96	1.90	0.20
*Fat*	2.27	0.97	1.14	0.59	0.31	0.04	0.29	0.03	0.28	0.04	0.34	0.08
*Muscle*	1.62	0.19	2.66	0.55	0.89	0.11	1.12	0.17	0.79	0.08	1.02	0.06
*Bone*	3.94	0.50	5.28	1.49	3.28	0.25	4.10	0.37	2.71	0.23	4.84	0.53
*Bladder*	20.49	6.42	7.51	0.45	3.81	0.27	3.68	0.43	3.96	0.30	5.93	1.06
*Stomach*	9.37	0.30	4.84	1.52	2.48	0.37	0.99	0.09	2.52	0.27	1.36	0.13
*Brain*	0.55	0.12	1.30	0.69	0.19	0.02	0.19	0.02	0.19	0.02	0.23	0.01
*Tumor*	6.92	0.63	3.15	1.04	3.61	0.34	3.72	0.43	2.96	0.37	1.44	0.14

*Notes:* *Ex vivo* biodistribution in NSG mice bearing STC-1, COLO-320 and PC12 xenografts after injection with 1.5MBq (40μCi) of D-[^18^F]FDOPA; mice were subsequently sacrificed at 30min and 120min p.i. time points for tissue retrieval; average values and standard errors (SEM) have been shown for each tissue.

In support of the obtained PET images, *ex vivo* biodistribution results also indicated significant off target L-[^18^F]FDOPA uptake in the pancreas, small intestines, stomach, kidneys, spleen, and liver, with the pancreas being the largest sink site, even 120min post tracer injection. At the 30min post injection time point, pancreatic uptake of L-[^18^F]FDOPA in STC-1, PC12 and COLO-320 mouse models was 44.22±1.93%ID/g, 50.08±2.08%ID/g and 57.82±1.40%ID/g, respectively, decreasing to 20.71±0.89%ID/g, 36.66±3.81%ID/g and 37.13±5.43%ID/g, after 120min. The primary non-specific retention site for D-[^18^F]FDOPA was also the pancreas followed by kidneys, spleen kidneys, stomach, and small intestines. However, retention in these tissues was generally much lower compared to levels observed for L-[^18^F]FDOPA, particularly at the 120min time point. Pancreatic retention of D-[^18^F]FDOPA in STC-1, PC12 and COLO-320 mice at 30min was 61.64±10.01%ID/g, 28.22±4.35%ID/g and 34.93±6.63%ID/g, respectively, decreasing to 9.75±0.36%ID/g, 3.42±0.31%ID/g and 3.12±0.68%ID/g, at 120min. The faster clearance rate of D-[^18^F]FDOPA was most evident at 120min in COLO-320 mice, illustrating a 12-fold lower pancreatic retention compare to L-[^18^F]FDOPA.

The second site of highest off-target tracer retention was the small intestines. The non-specific L-[^18^F]FDOPA intestinal retention in STC-1, PC12 and COLO-320 mice at 30min was 16.04±2.47%ID/g, 11.50±1.34%ID/g and 15.72±1.28%ID/g, respectively, and 5.08±0.10%ID/g, 10.02±0.91%ID/g and 8.66±0.63%ID/g, at 120min. The significant and persistent retention of L-[^18^F]FDOPA was 5-fold and 3-fold higher than tumor uptake levels in COLO-320 mice at 30min and 120min, respectively. In contrast, D-[^18^F]FDOPA intestinal retention in STC-1, PC12 and COLO-320 mice was 10.28±1.41%ID/g, 3.76±0.96%ID/g and 3.89±1.98%ID/g, respectively, at 30min, and 3.80±0.67%ID/g, 1.90±0.20%ID/g and 1.46±0.34%ID/g, at 120min. Similar results were obtained for other non-specific tissues further supporting superior and faster clearance kinetics of D-[^18^F]FDOPA. Another interesting finding from the *ex vivo* biodistribution studies was that while L-[^18^F]FDOPA was consistently retained in all extracted mouse brain tissues (average 2.21±0.17%ID/g at 30min and 1.22±0.16%ID/g at 120min in all NET mice), D-[^18^F]FDOPA brain accumulation was negligible in all cases (average 0.31±0.05%ID/g at 30min and 0.34±0.08%ID/g at 120min in all NET mice), supporting a modified metabolic/uptake mechanism for these enantiomers in the brain.

## DISCUSSION

While L-[^18^F]FDOPA has increasingly become an important tool for detection of NETs, its diagnostic sensitivity is hampered by its physiological uptake in the striatum, kidneys, pancreas, kidneys, liver, gallbladder, biliary tract, esophagus, myocardium, duodenum, and adrenal glands. In this report, we carried out a side-by-side evaluation of L-[^18^F]FDOPA and its enantiomeric isomer, D-[^18^F]FDOPA, in five NET cell lines and three NET mouse models. Our results demonstrate multiple key findings.

The first consistent observation was the significant variability in the biodistribution of each tracer among the studied NET mouse models. For instance, L-[^18^F]FDOPA uptake in COLO-320 and STC-1 tumors was >2 fold higher in the latter. These results suggest different levels of amino acid metabolism and tracer retention mechanisms between NETs. In addition, L-[^18^F]FDOPA clearance was also different among the NET models. For instance, pancreatic retention of this tracer remained at 37±5.43%ID/g in COLO-320 and 36.66±3.81%ID/g in PC12 120min p.i., while it was reduced to 20±0.89%ID/g in STC-1 mice. A similar observation was made in the small intestines with 10.01±0.91%ID/g uptake in PC12, 8.66±0.63%ID/g in COLO-320, and a lower 5.08±0.10%ID/g uptake in STC-1 mice at the same time point. While the mechanisms for L-[^18^F]FDOPA pancreatic accumulation are not yet fully understood, literature reports have consistently shown a reduction in tracer pancreatic uptake levels following advanced administration of oral Carbidopa [33],[34],[25]. Considering that inhibition of AADC-mediated conversion of L-[^18^F]FDOPA to [^18^F]fluorodopamine results in lowered pancreatic uptake, it can be reasonably hypothesized that variations in AADC activity between NETs, as well as differences in amino acid metabolism and storage, would likely be key contributors to the observed variability of L-[^18^F]FDOPA pancreatic uptake in NET subtypes. In support of this argument, AADC activity has also been shown to be important for the intracellular retention of decarboxylated L-[^18^F]FDOPA, which may also explain false-negative findings in detection of insulinomas and hyperplastic β-cell islets when using carbidopa [35], [36]. In line with those observations, variability of pancreatic L-[^18^F]FDOPA uptake in our studies is likely owing to the disease-dependent metabolism of this tracer and the availability of AADC in peripheral tissues.

The observed amino acid metabolic differences between NETs were also replicated by D-[^18^F]FDOPA. However, interestingly and contrary to the trends observed for [^18^F]FDOPA, D-[^18^F]FDOPA showed higher non-specific retention in STC-1 mice over 120min post tracer administration. Although, biodistribution of [^18^F]FDOPA enantiomers were not previously addressed until this report, our findings are still in line with published studies showing variable amino acid requirements among NET subtypes [37]. For instance, pheochromocytomas process/metabolize higher levels of amino acids, compared to other NETs, leading to elevated secretion of the catecholamines, metanephrine and normetanephrine, to such an extent that plasma metanephrine levels are currently used as a sensitive diagnostic indicator for this malignancy [38]. Our findings further emphasize such metabolic differences between NETs and discourage the use of a single universal PET tracer for all NET subtypes.

The second key observation was that all biological evaluation, *in vitro*, *in vivo*, and *ex vivo*, generally indicated higher NET uptake of L-[^18^F]FDOPA, compared to D-[^18^F]FDOPA, with the exception of COLO-320 tumors at the 120min time point, where no statistically significant differences were noted between the two tracers. Interestingly, while the highest L-[^18^F]FDOPA NET uptake among the studied models was in PC12 tumors (pheochromocytomas), the highest D-[^18^F]FDOPA uptake was observed in STC-1 tumors (intestinal NETs). These observations imply a difference either in LAT1-mediated transport of D- versus L-FDOPA and/or the route of D/L-FDOPA enzymatic metabolism that leads to varying cellular retention levels. Literature reports demonstrate that following its administration, D-DOPA is unidirectionally converted to dihydroxyphenylpyruvic acid (DHPPA) by the enzyme D-amino acid oxidase (DAO), which then, via an alternative dopamine biosynthesis pathway, is transaminated from the α-keto acid to L-DOPA [39-42]. The multi-step nature of this route makes for a slower process as illustrated by the longer retention of D-DOPA metabolites in the brain [43]. As a result, because of its slow nature, the contribution of *in vivo* D-DOPA to L-DOPA conversion to overall tracer uptake and retention, while certainly present, is unlikely to be a major factor to the biodistribution of D-[^18^F]FDOPA. Therefore, the observed D-[^18^F]FDOPA pharmacokinetics are most likely a result of transporter activity in combination with amino acid metabolism requirements in each NET model. In support of this argument and our findings in this report, LAT1 has been shown to be very tolerant of modified amino acid analogues and is also non-stereoselective, transporting both D- and L-amino acids, including the DOPA enantiomers (IC_50_ = 69±29μM for L-DOPA and 46±23μM for D-DOPA) [44-47]. Therefore, the lower overall tumor retention of D-[^18^F]FDOPA is most likely, and primarily a result of its intracellular metabolism/processing rather than its LAT1-mediated transport. Similar to L-[^18^F]FDOPA, which requires specific metabolic processing (e.g. AADC-mediated decarboxylation) for enhanced cellular retention and accumulation, D-[^18^F]FDOPA may also have such requirements to be retained and accumulated in NET cells. It is therefore very likely that the lack of such events, in combination with metabolic differences among NETs, is a major determinant for the observed D-[^18^F]FDOPA tumor retention levels in the studied NET models. However, it is also because of the lack of those metabolic events that D-[^18^F]FDOPA is likely not as extensively metabolized, leading to the observed enhanced clearance from non-specific tissues, when compared to L-[^18^F]FDOPA.

In line with the above argument, the overall clearance kinetics of D-[^18^F]FDOPA were much faster than L-[^18^F]FDOPA, showing the highest tumor uptake levels at 30min post tracer administration, followed by significant systemic clearance within 120min p.i. More importantly, D-[^18^F]FDOPA exhibited markedly lower non-specific retention, compared to L-[^18^F]FDOPA in off-target organs, followed by further clearance 120min post tracer administration. Pancreatic retention of D-[^18^F]FDOPA, compared to L-[^18^F]FDOPA, in STC-1, COLO-320 and PC12 tumors was approximately 2, 12, and 11 fold lower at 120min p.i., respectively. Retention of D-[^18^F]FDOPA in small intestines was also reduced in all NET models, being up to 4 fold lower than L-[^18^F]FDOPA at 30min and up to 7 fold lower at 120min p.i. Similar observations were made for renal and hepatic retention of D-[^18^F]FDOPA, which were up to 4 fold lower than that with L-[^18^F]FDOPA. These results further support and suggest a reduced *in vivo* metabolism of D-[^18^F]FDOPA, leading to decreased off-target tissue retention and enhanced overall clearance, when compared to L-[^18^F]FDOPA.

As initially hypothesized, our results illustrated lower retention and enhanced clearance of D-[^18^F]FDOPA from off-target tissues. This is particularly beneficial in diagnosis of cancers in tissues with inherently high L-[^18^F]FDOPA physiological uptake, which limit the diagnostic applications of this clinical tracer. In addition, the faster systemic clearance of D-[^18^F]FDOPA can reduce radioactivity exposure levels to patients with NETs, as L-[^18^F]FDOPA remains in peripheral tissues long after completion of the PET scan acquisition. However, the superior clearance of D-[^18^F]FDOPA does come at the cost of overall tumor uptake levels as D-[^18^F]FDOPA exhibits lower tumor retention values compared to L-[^18^F]FDOPA in all studied NETs, particularly at earlier time points post tracer administration. As such, the diagnostic applications of D-[^18^F]FDOPA will be limited to malignancies that present diagnostic challenges for L-[^18^F]FDOPA owing to its physiological uptake. These findings also emphasize the importance of proper selection of imaging time points for patients with NETs for achieving optimal tumor uptake as well as clearance from non-specific tissues. In addition, our results further support the notion of precision medicine as they clearly demonstrate that the use of a single PET imaging agent is not optimal for detection of all NET subtypes. Our group is currently working on evaluating D-[^18^F]FDOPA in animal models with NETs exhibiting a more closely matched genomic signature to those observed in humans for a more clinically-relevant assessment of the inherent differences in the biodistribution and metabolism of these tracers.

## MATERIALS AND METHODS

Cassettes and reagent kits for radiotracer production were obtained from Trasis (Ans, Belgium). Anti-LAT1 antibodies were purchased from Cell Signaling Technology (MA, USA), Santa Cruz Biotechnology (Texas, USA), and Novus Biologicals (CO, USA). All cell culture reagents were purchased from Invitrogen unless otherwise noted.

### Preparation of [^18^F]FDOPA enantiomers

Both [^18^F]FDOPA analogues were synthesized using an AllinOne Trasis PET Tracer Synthesizer following established literature methods [28]. Crude reaction mixtures were purified by semi-preparative high-performance liquid chromatography The HPLC-purified product underwent a second purification by chiral RP-HPLC for separation of the D and L-enantiomers and to ensure maximal enantiomeric excess. Final products were analyzed by analytical chiral HPLC for radiochemical and enantiomeric purity. The analytical RP-HPLC system included an Agilent 1260 Infinity System equipped with a quaternary pump, an HiP ALS autosampler, a DAD UV detector and a Bioscan Flow-Count interface with a NaI radioactivity detector. For analytical RP-HPLC an Atlantis® T3, 5μm, 4.6×100mm column was used with 10mM phosphate buffer (pH 2.2) as the eluent and a flow rate of 2mL/min. The chiral chromatography system included a Waters 484 Tunable Absorbance Detector, a Gabi Raystar Radioactivity detector and a Waters 515 HPLC pump. Chiral chromatography was carried out using a Daicel CR(+) Crown Pak, 5μm, 4.0x150mm column and a pH 2 perchlorate buffer as the eluent with a flow rate of 1mL/min. Semi-preparative RP-HPLC, an integrated component of the Trasis AllinOne system, utilized an XBridge® BEH Shield RP18, 5μm, 10x250mm column with an acetate buffer containing sodium ascorbate as the eluent and a flow rate of 5mL/min. Chromatographic data were acquired and analyzed with Agilent OpenLAB chromatography data system (Rev. A.04.02). Radioactivity measurements were made using a Comecer model TALETE HC dose calibrator.

### Flow cytometry

One million cells were stained on ice for 20 min with either 5μl of LAT1-FITC (Novus Biologicals) or 5μl of 7-AAD (BD Pharmingen) to exclude dead cells, in 0.5% BSA/ 2mM EDTA/ PBS. Samples were run on a BD FACSCalibur system (Becton Dickinson). Generated data were analyzed using FlowJo software (FlowJo, LLC). Cells were analyzed on a BD LSR II flow cytometer with BD FACSDiva Software (BD Biosciences).

### Cell lines

All NET cell lines were purchased from the American Type Culture Collection. TGP61 and TGP52 cell lines were maintained in a 1:1 mixture of Dulbecco's modified Eagle's medium (DMEM) and Ham's F12 medium, with 10% heat-inactivated FBS. PC12 cells were maintained in RPMI-1640 medium with 10% heat-inactivated horse serum and 5% FBS. STC-1 cells were maintained in DMEM medium with 10% FBS. COLO-320 cells were maintained in RPMI-1640 medium with 10% FBS. All cell lines were maintained in a humidified incubator under 5% CO_2_ at 37°C.

### *In vitro* binding assays

*In vitro* binding assays were carried out, in triplicate, in PBS with 0.5% FBS, for 30mins at ambient temperatures using 1 million cells and repeated three times. Blocking studies were carried out using 10meq of the commercial LAT1 inhibitor, 2-Aminobicyclo[2.2.1]heptane-2-carboxylic acid (TOCRIS, MN, USA). Following incubation with each tracer, cells were rinsed three times with cold PBS prior to being counted on an automated gamma counter (1282 Compugamma CS; Pharmacia/LKBNuclear, Inc.) along with external standards. Obtained counts were converted into percentage incubated dose per million cells based on normalization to external standards. Experiments were performed in triplicate and repeated three times.

### Mouse models

All animal studies were carried out according to regulations set forth and approved by the Johns Hopkins Animal Care and Use Committee. Female, 6- to 8-weeks-old immunodeficient NOD *scid gamma* (NSG) mice were subcutaneously inoculated with 1 million PC12, STC-1 or COLO-320 cells in 100 mL of Hank’s balanced salt solution in the top flank. Tumors of 4–6 mm in diameter (typically 14-21 days post inoculation) were utilized for *in vivo* imaging or *ex vivo* biodistribution studies.

### *In vivo* PET-CT imaging

Mice (n=4) were injected with 9MBq (250μCi) of each tracer in 150μL of saline intravenously and anesthetized with 3% isoflurane prior to being placed on the scanner bed. Mice were kept warm with an external light source and maintained at 1% isoflurane levels while being scanned. PET imaging (2 beds; 10 min per bed) was carried out using an ARGUS small-animal PET/CT scanner (Sedecal). A CT scan (512 projections) was obtained at the end of each PET scan for anatomical co-registration. For *in vivo* blocking experiments, mice were injected with 10meq of LAT1 inhibitor. PET data were reconstructed using the 2-dimensional ordered subsets-expectation maximization algorithm (2D-OSEM) and corrected for dead time and radioactive decay. Data visualization and image generation were carried out using Amira 2019.1 (ThermoFisher Scientific).

### PET image analysis

Quantitative *in vivo* image analysis was carried out in AMIDE (SourceForge) 1.0.4 utilizing ellipsoid region-of-interest volume integrations with 3 slices entailing a depth of 1.9 mm per slice. The %ID per cc values were calculated based on a calibration factor obtained from a phantom (known radioactive quantity in a known volume).

### *Ex vivo* biodistribution

Mice (n=5 per group) were intravenously injected with 1.5MBq (40μCi) of each tracer in 150μL of saline. *Ex vivo* biodistribution studies were performed at 30min and 120min time points after tracer administration. Blood, liver, spleen, heart, lungs, kidneys, small intestines, stomach, muscle, fat, bone, bladder, brain and tumor tissues were retrieved, weighed, and counted in an automated gamma counter (1282 Compugamma CS). Percentage injected dose per gram (%ID/g) values were calculated based on normalization to external standards, counted in triplicate, and accounted for signal-decay correction.

### Western blot analysis of tumor tissues

Frozen xenografts were pulverized and homogenized in ice-cold RIPA buffer containing protease and phosphatase inhibitors (Roche, #1183615300 and #14906845001) with a mortar and pestle. 40μg of extracted protein was vertically electrophoresed on 4-12% Bis-Tris NuPage Novex Gel in MOPS SDS running buffer (Invitrogen), then transferred to Hybond C Extra membrane (GE Healthcare). Membranes were stained with Ponceau stain to confirm protein transfer, then blocked with 5% powdered milk in PBS with 0.2% Tween-20 (PBST) for one hour. Membranes were probed with primary antibody in 5% milk/PBST at 4°C overnight, rinsed with PBST, then probed with secondary antibody (GE Healthcare) at 1:2000 dilution in 5% milk/PBST for 1h. After rinsing with PBST, membranes were treated with ECL Plus Detection Reagent (GE Healthcare) for 1 minute and subsequently exposed to a Hyblot CL autoradiography film to determine protein expression. Antibodies to LAT1 (Santa Cruz Biotechnology, D10, sc-374232) and β-actin (#A1978, Sigma) or GAPDH (#MAB374, Millipore) were used. Quantitation was done using ImageJ software.

### Immunohistochemistry

Where applicable, retrieved tumors were evaluated by hematoxylin and eosin staining and, for LAT1 expression, immunohistochemistry (IHC). Harvested tissues were fixed in 10% buffered formalin and embedded in paraffin before being sectioned with a 5μm thickness. After deparaffinizing, antigen retrieval was performed using 10mM citrate buffer, pH 6 (Dako) for 20 min in a steamer. Endogenous peroxidase activity was quenched using peroxidase block (BLOXALL, Vector) for 10 min. Tumor sections were blocked with normal serum as per the Vectastain elite staining kit (Vector) instructions. Sections were incubated overnight at 4°C with the 1:100 diluted primary anti-LAT1 antibody (Novus Biologicals). Slides were subsequently washed and incubated with biotinylated secondary antibody for 30 min, washed again and incubated with streptavidin for 30 min per Vectastain elite kit instructions. Staining with 3’3-diaminobenzidine (DAB) was carried out according to the manufacturer protocols (Vector). Sections were counterstained with Gill’s hematoxylin followed by dehydration with gradient alcohol and xylene washes prior to mounting with a cover slip.

### Statistical analysis

Statistical analysis of *in vitro* and *ex vivo* biodistribution data was carried out in GraphPad Prism 7.04 (GraphPad Software, Inc.) and Excel 2016 (Microsoft). An unpaired 2-tailed t-test was used for determination of statistical significance and set when the p value was less than 0.05.

## Abbreviations

PET: positron emission tomography
CT: computed tomography
NET: neuroendocrine tumor
NEN: neuroendocrine neoplasm
PRRT: peptide receptor radionuclide therapy
SSTR2: somatostatin receptor 2
L-DOPA: 3,4-dihydroxy-L-phenylalanine
D-DOPA: 3,4-dihydroxy-D-phenylalanine
L-[^18^F]FDOPA: 3,4-dihydroxy-6-[^18^F]fluoro-L-phenylalanine
D-[^18^F]FDOPA: 3,4-dihydroxy-6-[^18^F]fluoro-D-phenylalanine
LAT1: L-type amino acid transporter
MTC: medullary thyroid cancer
%ID/g: percent injected dose per gram
%ID/cc: percent injected dose per cubic centimeter
p.i.: post injection
DAO: D-amino acid oxidase
DHPPA: dihydroxyphenylpyruvic acid
PBST: phosphate buffered saline with tween 20
DMEM: dulbecco’s modified eagle medium.
